# Design and Application of a Low pH Upflow Biofilm Sulfidogenic Bioreactor for Recovering Transition Metals From Synthetic Waste Water at a Brazilian Copper Mine

**DOI:** 10.3389/fmicb.2018.02051

**Published:** 2018-08-30

**Authors:** Ana L. Santos, D. Barrie Johnson

**Affiliations:** College of Natural Sciences, Bangor University, Bangor, United Kingdom

**Keywords:** biofilms, bioreactor, *Firmicutes*, metal removal, sulfate-reducing bacteria, sulfidogenesis, transition metals

## Abstract

A sulfidogenic bioreactor, operated at low pH (4–5), was set up and used to remove transition metals (copper, nickel, cobalt, and zinc) from a synthetic mine water, based on the chemistry of a moderately acidic (pH 5) drainage stream at an operating copper mine in Brazil. The module was constructed as an upflow biofilm reactor, with microorganisms immobilized on porous glass beads, and was operated continuously for 462 days, during which time the 2 L bioreactor processed >2,000 L of synthetic mine water. The initial treatment involved removing copper (the most abundant metal present) off-line in a stream of H_2_S-containing gas generated by the bioreactor, which caused the synthetic mine water pH to fall to 2.1. The copper-free water was then amended with glycerol (the principal electron donor), yeast extract and basal salts, and pumped directly into the bioreactor where the other three transition metals were precipitated (also as sulfides), concurrent with increased solution pH. Although some acetate was generated, most of the glycerol fed to the bioreactor was oxidized to carbon dioxide, and was coupled to the reduction of sulfate to hydrogen sulfide. No archaea or eukaryotes were detected in the bioreactor microbial community, which was dominated by acidophilic sulfate-reducing *Firmicutes* (*Peptococcaceae* strain CEB3 and *Desulfosporosinus acididurans*); facultatively anaerobic non-sulfidogens (*Acidithiobacillus ferrooxidans* and *Actinobacterium* strain AR3) were also found in small relative abundance. This work demonstrated how a single low pH sulfidogenic bioreactor can be used to remediate a metal-rich mine water, and to facilitate the recovery (and therefore recycling) of target metals. The system was robust, and was operated empirically by means of pH control. Comparison of costs of the main consumables (glycerol and yeast extract) and the values of the metals recovered showed a major excess of the latter, supporting the view that sulfidogenic biotechnology can have significant economic as well as environmental advantages over current approaches used to remediate mine waters which produce secondary toxic wastes and fail to recover valuable metals.

## Introduction

The pollution of water bodies caused by the oxidative dissolution of sulfide minerals in rock strata is a global occurrence. Where this is a natural phenomenon, such as sites in the high Arctic and Antarctica (Langdahl and Ingvorsen, 1997; Dold et al., 2013) it is referred to as “acid rock drainage” (ARD), though far more prevalent are water bodies that have been impacted by the mining of metals and coals (“acid mine drainage,” AMD; Blowes et al., 2014). The process whereby ARD/AMD forms may be entirely chemical, though it is greatly accelerated by diverse species and consortia of mostly lithotrophic (“rock eating”) bacteria and archaea in the presence of both air and water (Blowes et al., 2014). Acid mine drainage may form within mine wastes (tailings and rock dumps) that are impacted with surface water or in underground workings (groundwater), and its production can continue long after mines are decommissioned. The major issues of concern with AMD are chiefly its acidity (both proton and mineral acidity, though mine waters can also contain significant bicarbonate alkalinity), elevated concentrations of soluble metals (iron and other transition metals, and aluminum) and metalloids (such as arsenic), high concentrations of sulfate, and large osmotic potentials, all of which can negatively impact the biota of water bodies that are impacted by AMD (Blowes et al., 2014).

Various approaches can be used to remediate AMD (reviewed in Johnson and Hallberg, 2005). The most widely used of these is addition of alkalizing materials such as lime (CaO) to raise the pH of the water while simultaneously aerating the water to promote the oxidation of relatively soluble ferrous iron to ferric iron, which is highly insoluble above pH ∼2.5 (iron is frequently the most abundant transition metal present in AMD). This produces a metal carbonate/hydroxide and gypsum-rich material which can be compressed to form a “high density sludge” (e.g., Coulton et al., 2002). Also in widespread use are constructed wetlands. These use processes that occur in aerobic and/or anaerobic ecosystems to attenuate mine waters, ideally immobilizing and accumulating metals and metalloids within surface ochre deposits or subsurface composts (Younger et al., 2003). However, both of these approaches (often referred to as active and passive remediation, respectively) have major drawbacks which include: (i) high costs both in terms of construction and (especially in the case of chemical treatment) operation; (ii) the production of hazardous secondary waste materials (metal-rich sludges or metal-rich spent composts that require long-term storage in designated landfill sites, posing the potential risk that highly toxic elements will be remobilized), and (iii) metals of value present in the treated waters are not recovered and recycled. Both active chemical and passive wetland remediation mitigation practices have major drawbacks and should be considered only as intermediary solutions to the problem until more environmentally acceptable solutions have been developed and validated. Active biological mitigation using bioreactor modules is one such alternative. Both oxidative and reductive laboratory-scale bioreactors that operate at low pH values have been developed for this purpose (Johnson, 2014), and also used in tandem to mitigate and recover metals from complex mine waters (e.g., Hedrich and Johnson, 2014). Industrial-scale sulfidogenic bioreactors have also been used for remediating mine waters, though these use neutrophilic sulfur- and sulfate-reducing bacteria that need to be shielded from direct contact with acidic mine waters (Boonstra et al., 1999; van Houten et al., 2006, 2009).

Brazil is the world’s 15th largest producer of copper concentrates. The mining company Vale S.A. is responsible for the majority of the production, operating at the Sossego and Salobo copper mines. At the Sossego mine, moderately acidic AMD flows continuously from an ore deposit along a channel in the soil to a run-off area. Currently, the drainage is treated with limestone along the course of the channel to increase its pH and precipitate heavy metals (Pereira et al., 2014; Ñancucheo et al., 2017). Even though this is effective in removing metals and increasing water pH, it has the inherent disadvantages that accompany active chemical treatment, described above. Here we describe an alternative approach using a single minimally engineered and robust sulfidogenic bioreactor that facilitates the recovery of copper and other transition metals from mine water at the Sossego mine, in an approach that is both environmentally benign and cost-effective.

## Materials and Methods

### Preparation and Chemical Composition of Synthetic Mine Water

**FIGURE 1:**
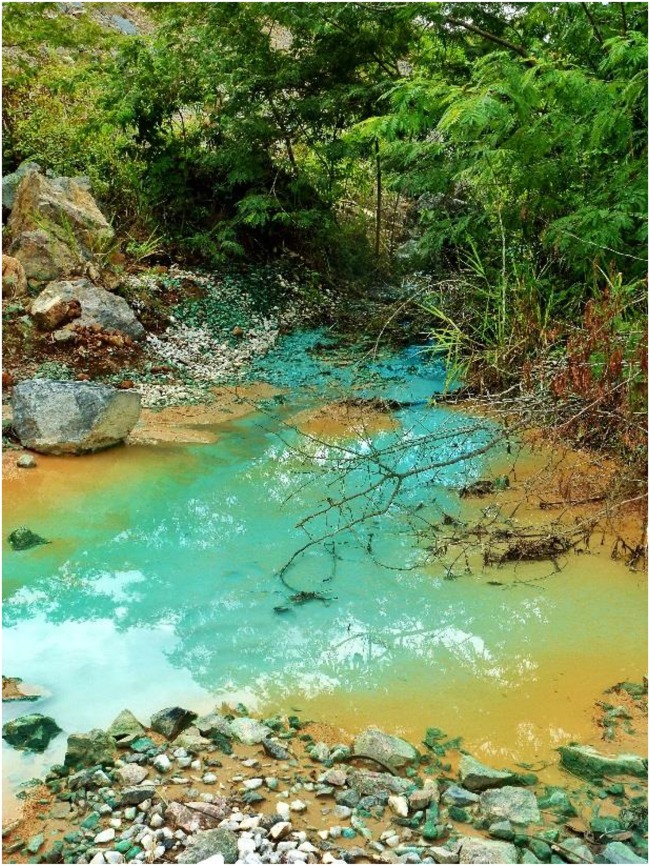
Image of the moderately acidic metal-rich stream at the Sossego mine, Brazil (courtesy of the Instituto Tecnológico Vale (ITV), Belem, Brazil).

Synthetic AMD, based on the chemical composition of a copper-rich waste stream (**Figure 1**) at the Sossego copper mine at Canaã dos Carajás, Brazil (6° 26′ 25.625″ S 50° 2′ 03.068″ W) was prepared in the Bangor laboratories using analytical data provided by the Vale Technological Institute, Belém, Brazil. The AMD at the Sossego mine had a pH of 5.0, a redox potential (*E*_H_) of +270 mV and a conductivity of ∼3 μS cm^-1^. The dominant dissolved transition metal present was copper, and it also contained smaller concentrations of cobalt, nickel, and zinc, but due to its relatively high pH, iron concentrations were very low (∼2 μM). Basal salts (Ñancucheo et al., 2016) were added to the synthetic mine water, together with glycerol (5 mM, as the principal electron donor) and 0.1 g yeast extract L^-1^ (to supply growth factors) and its pH adjusted to 5.0. The chemical composition of the amended synthetic mine water, and comparison with AMD at the Sossego mine, is shown in **Table 1**.

**Table 1:** Chemical compositions of AMD at the Sossego mine and the amended synthetic version.

Analyte	Sossego AMD	Synthetic AMD
Al^3+^	0.04	<
As_total_	<0.01	<
Ca^2+^	7.03	7.06
Cd^2+^	<0.01	<
Cl^-^	0.04	0.71
Co^2+^	0.04	0.04
Cr^3+^	<0.01	<
Cu^2+^	7.50	7.50
F^-^	0.53	<
Fe^2+^	<0.05	<
Fe^3+^	0.15	<
K^+^	0.28	0.95
Mg^2+^	4.01	6.00
Mn^2+^	0.15	0.15
N_total_	1.20	7.92^∗^
Na^+^	2.10	3.04
Ni^2+^	0.25	0.25
P_total_	<0.01	0.37
Pb	<0.01	<
SO_4_^2-^	15.68	26.47
Zn^2+^	0.02	0.02
DOC^∗∗^: theoretical/measured (mg L^-1^)	n.d.	230/234


*Basal salts, glycerol and yeast extract were added only to partly processed (copper-free) synthetic AMD. Concentrations are expressed in mM, unless indicated otherwise. n.d. not determined; < indicates elements that were not added directly to the synthetic AMD; ^∗^includes 1 mM estimated to be present in yeast extract; ^∗∗^dissolved organic carbon.*

### Commissioning and Operation of the Low pH Sulfidogenic Bioreactor (aSRBR)

**FIGURE 2:**
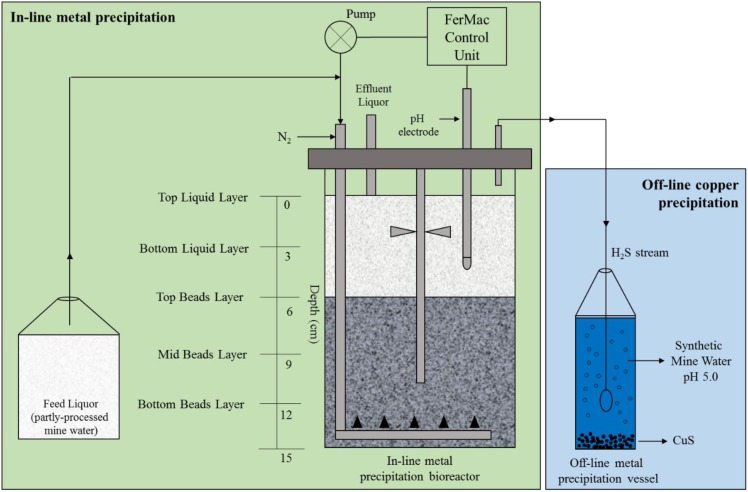
Schematic representation of the low pH sulfidogenic bioreactor (aSRBR). Excess hydrogen sulfide produced in the aSRBR was delivered to an off-line vessel, containing initially copper sulfate and later synthetic mine water, in order to mediate selective removal of copper. Following copper precipitation, the more acidic partly processed water was used as feed liquor for the bioreactor.

A 2 L (working volume) upflow biofilm bed sulfidogenic bioreactor, based on similar modules described by Ñancucheo and Johnson (2012) and Santos and Johnson (2017), was commissioned for these experiments (**Figure 2**). In brief, acidophilic sulfate-reducing bioreactor (aSRBR) and other bacteria immobilized on 1–2 mm diameter porous beads manufactured from recycled glass (by Dennert Poraver GmbH, Schlûsselfeld, Germany) were removed from a 5 L continuous flow “mother reactor” to act as the inoculum for the new bioreactor. The “mother reactor” had been inoculated several years previous to this with pure cultures of *Desulfosporosinus acididurans* (a moderately acidophilic sulfate-reducer) and *Acidocella aromatica* (a non-sulfidogen; Kimura et al., 2006), and an enrichment culture from a sulfidogenic mat in a drainage channel at an abandoned copper mine in Spain (Cantareras; Rowe et al., 2007). The biofilm bed (∼1.3 L) was covered with ∼700 mL of amended copper-free synthetic AMD, leaving a surface air space of ∼250 mL. The bioreactor top plate was attached and the vessel gassed continuously with ∼150 mL min^-1^ of oxygen-free nitrogen, which acted as a carrier gas to remove and transfer H_2_S generated in the bioreactor. Influent liquors were pumped into the bottom of the bioreactor vessel *via* a perforated L-tube, and percolated upward through the biofilm bed into the overlying liquid layer which was stirred gently at 50 rpm to ensure its homogeneity. The volume of liquid in the bioreactor was maintained at 2 L using a fixed depth hollow tube inserted through the top plate connected to a pump operated in continuous flow mode. Flow rates of influent liquors (and therefore hydraulic retention times; HRTs) were dictated by the pH differential of the influent liquor and the set operating pH of the bioreactor. At low pH, microbial reduction of sulfate to hydrogen sulfide is net consumptive of hydronium ions and therefore the rate at which this occurred was used to regulate the flow of influent liquor, using a pH electrode coupled to a pump. This necessitated the pH of the influent liquor to be lower than that at which the bioreactor was operated. For most experiments, the latter was set at 4.0 and the temperature at 30°C, though this was later changed to pH 5.0 and 35°C, as described below.

Following setting up the aSRBR it was primed for 44 days using an influent liquor containing 2.5 mM glycerol, 0.1 g yeast extract L^-1^, 5.0 mM K_2_SO_4_, 100 μM FeSO_4_⋅7H_2_O and basal salts/trace elements (Ñancucheo et al., 2016) adjusted initially to pH 2.5. The aSRBR was connected to a 500 mL off-line vessel containing 100 mL of 25 mM CuSO_4,_ and changes in soluble copper concentrations used to determine the rate of production of H_2_S by the aSRBR. This phase continued until a relatively constant rate of H_2_S production was obtained.

### Combined Off-Line and In-Line Removal of Transition Metals From Synthetic AMD

The pH of Sossego mine water and its synthetic equivalent (pH 5.0) was too high for it to be pumped directly into the aSRBR, which usually operate at between pH 2.5 and 5.0. In addition, although it would have been possible to precipitate copper within the bioreactor vessel, this was deemed inappropriate since its high concentration in the AMD would have caused void spaces in the bioreactor vessel to become rapidly infilled with copper sulfide precipitates. To circumvent these problems, the aSRBR was configured to over-produce H_2_S (i.e., in excess of that required to precipitate cobalt, nickel, and zinc in-line) by increasing the glycerol concentration in the influent liquor from 2.5 to 5.0 mM, and the excess gas transferred to an off-line vessel where it contacted unamended synthetic AMD, selectively precipitating copper (as a sulfide phase) and causing the water to become far more acidic and therefore suitable as an influent liquor for the bioreactor. Copper-free synthetic AMD was then amended with basal salts, glycerol and yeast extract (as described above) and used as the feed for the aSRBR. This system was maintained in continuous operation for 462 days, during which time 2,025 L of synthetic AMD was processed through the same 2 L aSRBR module.

### pH and Molecular Analysis of the Biofilm Bed

During most of the time that tests were carried out with the aSRBR, only liquid samples from the surface layer in the bioreactor vessel or the effluent liquors were analyzed chemically and for their microbial compositions, in order not to disrupt the biofilm bed. At the end of the experiment (when the bioreactor was being maintained at pH 5.0 and 35°C) a coring device was used to remove samples (5–10 cm^3^) at different depths within the biofilm bed (**Figure 2**). These were referred to as “top beads” (0–3 cm below the biofilm bed surface), “mid beads” (3–6 cm), and “bottom beads” (6–9 cm). The pH of the interstitial liquids were measured directly in these samples, after which the beads were centrifuged at 4,000 *g* for 10 min, the liquid phases discarded and the compositions of the microbial communities attached to the beads examined. DNA was extracted from the colonized beads using PowerSoil Ultraclean Microbial DNA Isolation Kits (MoBio, Carlsbad, CA, United States). At the same time, ∼20 mL of liquid from the surface of the aSRBR was filtered through a 0.2 μm nitro-cellulose filter membrane (Whatman, United Kingdom) and DNA extracted from that, again using a MoBio kit. Bacterial 16S rRNA genes were amplified, and analyzed using terminal restriction enzyme fragment length polymorphism (T-RFLP; Santos and Johnson, 2017). Terminal restriction enzyme fragments (T-RFs) were separated on a capillary sequencer (Beckman Coulter, CEQ8000) and identified by comparison to the database of acidophilic microorganisms maintained at Bangor University. The relative abundances of T-RFs were calculated from their peak areas. Attempts were also made to amplify archaeal 16S and eukaryotic 18S rRNA genes, using specific primers (Mariner et al., 2008; Kay et al., 2013).

### Scanning Electron Microscopy and EDAX Analysis

A bead sample (a mixture of all three layers) was visualized using scanning electron microscopy (SEM). The sample was coated with gold-palladium for 5 min immediately before being viewed with Zeiss Supra 40VP scanning electron microscope (Carl Zeiss, Germany). To provide elemental identification and semi-quantitative composition information of the samples, energy dispersive X-ray (EDAX) analysis was performed using an EDAX unit (Ametek Inc., United States) attached to the SEM.

### Analytical Techniques

The pH of filtered liquid samples was measured using a pHase combination glass electrode coupled to an Accumet 50 pH meter. Concentrations of transition metals, acetate, sulfate, and glycerol were determined using ion chromatography, as described by Santos and Johnson (2017). Concentrations of dissolved organic carbon were determined using a Protoc DOC analyser (Pollution and Process Monitoring Ltd., United Kingdom). Concentrations of soluble copper were measured using a colorimetric-based assay (Anwar et al., 2000). Bacteria were enumerated using a Thoma counting chamber and a Leitz Labolux phase-contrast microscope. Rates of hydrogen sulfide production were calculated from rates of off-line copper precipitation, since the stoichiometry of H_2_S produced to CuS generated is 1:1 (the solid phase product was confirmed by X-ray diffraction analysis to be covellite).

## Results

### Priming the Newly Commissioned aSRBR

**FIGURE 3:**
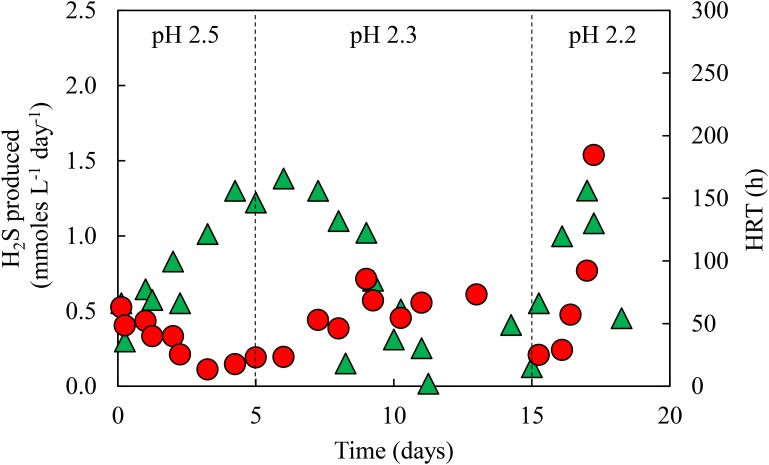
Changes in HRTs (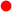) and rates of H_2_S production (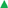) during priming of the aSRBR, where the pH of the influent liquor was lowered incrementally from 2.5 to 2.2.

Priming of the newly commissioned aSRBR continued for ∼20 days, by which time the bioreactor became adapted to receiving low pH (2.2) influent liquor, and rates of H_2_S production (determined from off-line precipitation of copper) fluctuated between 0.5–1.5 mmol L^-1^ day^-1^. **Figure 3** shows changes in HRTs and rates of H_2_S production during this period. Hydraulic retention times increased when the pH of the influent liquor was lowered, due to increasing concentrations of total acidity [both proton (H_3_O^+^) and bisulfate (HSO_4_^-^) acidity], but each time they subsequently decreased as the microbial community in the bioreactor adapted to the additional acid stress. Rates of H_2_S production followed the opposite trend, initially declining in response to increased acidity before subsequently increasing.

### Combined Off-Line and In-Line Removal of Metals From Synthetic AMD

**FIGURE 4:**
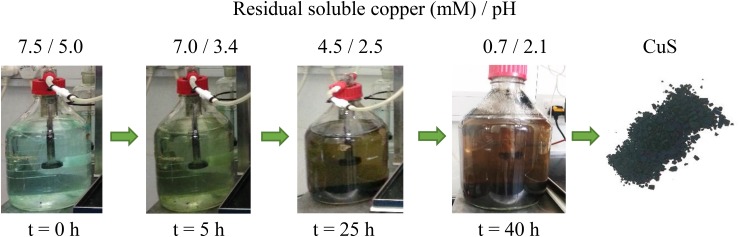
Time-lapse images of different stages of copper mineralisation in the off-line vessel. Analysis of the solid phase product (by XRD) confirmed that it was covellite (CuS).

At the end of the priming phase, the off-gas stream from the aSRBR was diverted into a 2 L Duran bottle that contained synthetic AMD which was not amended with nutrients or organic materials, as the reaction within the bottle was entirely abiotic. **Figure 4** shows time-lapse images of copper sulfide formation within the Duran bottle. After 48 h, >97% of the soluble copper had been removed and the pH of the synthetic mine water had fallen to 2.1 (Cu^2+^ + H_2_S + 2 H_2_O → CuS + 2 H_3_O^+^), making it suitable for use as influent liquor for the aSRBR. Following removal of solid-phase copper sulfide (by filtration through Whatman #1 filter paper), the partly processed synthetic AMD was sterilized (120°C, 20 min), glycerol, yeast extract and nutrient salts were added and the amended solution used as feed for the aSRBR.

**FIGURE 5:**
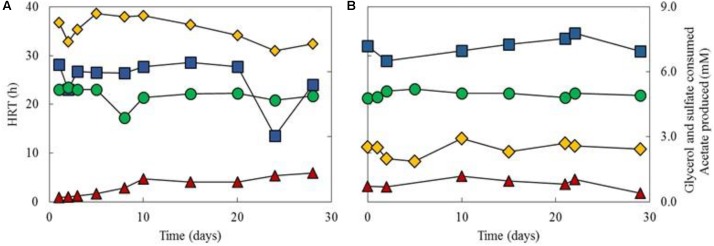
Performance data of the aSRBR during times when maintained at either pH 4.0 and 30°C **(A)**, or at pH 5.0 and 35°C **(B)**. Key: (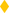) HRTs; (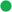) glycerol consumed (concentration in the influent liquor minus that in the effluent liquor); (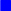) sulfate consumed; (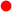) acetate produced. Concentrations of glycerol and sulfate in the influent liquor were 5 and 26.5 mM, respectively.

Performance data of the aSRBR during periods when it was maintained at either pH 4.0 and 30°C or at pH 5.0 and 35°C, are shown in **Figure 5**. Rates of sulfate reduction were 3.1 ± 0.96 and 14.8 ± 2.1 mmoles L^-1^ d^-1^ when the bioreactor was operated at pH 4.0 and 30°C, or at pH 5.0 and 35°C, respectively. Hydraulic retention times were initially >200 h when amended copper-free synthetic AMD was used as feed liquor, due in part to the lower pH (2.1) of this than the most acidic liquor (pH 2.2) used in the priming phase, and decreased to ∼50 h after 10 days. Throughout the 462 days that the aSRBR was in operation, most (>99%) of the 5 mM glycerol present in the feed liquor was metabolized, and acetate was detected in the effluent liquors at ∼1 mM. Concentrations of sulfate were also significantly lower in the effluent liquors (mean value of ∼19 mM) than in the influent liquor (26.5 mM). Numbers of microbial cells in the effluent liquors were typically about 6 × 10^7^ mL^-1^.

**FIGURE 6:**
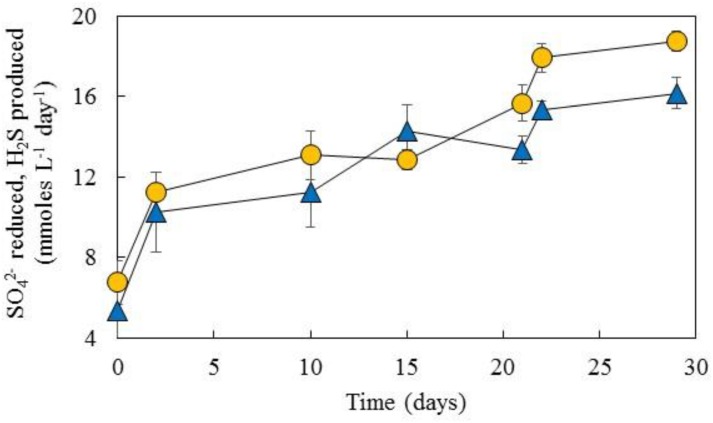
Comparison of net sulfate removal in the aSRBR (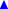) and theoretical production of hydrogen sulfide (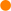, calculated from glycerol catabolism) during the period when the bioreactor was maintained at pH 5.0 and 35°C. The complete oxidation of glycerol (to CO_2_) generates 14 electrons/glycerol, and incomplete oxidation (to acetate + CO_2_) 6 electrons/glycerol. The dissimilatory reduction of sulfate to sulfide requires eight electrons. Data points depict mean values and error bars standard deviations of triplicated analyses of each sample.

Comparison of H_2_S production in this basis, i.e., calculated from glycerol catabolism and assuming zero fermentation of the alcohol, and sulfate removal during operation of the aSRBR is shown in **Figure 6**. The two line graphs were closely aligned, confirming that most of the glycerol used was coupled to sulfate reduction.

**FIGURE 7:**
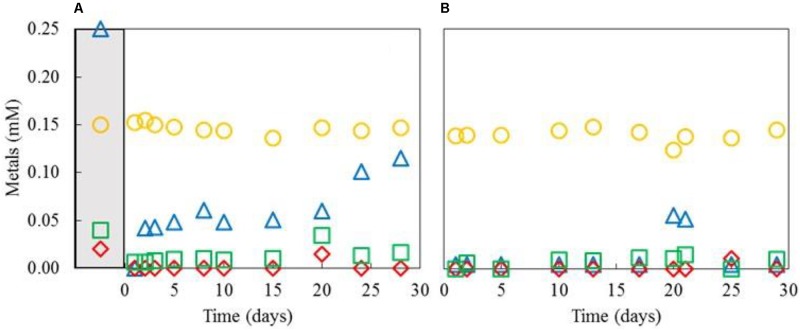
Concentrations of nickel (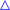), zinc (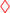), cobalt (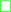), and manganese (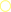) in effluent liquors from the aSRBR, when it was operated at either pH 4.0 and 30°C **(A)**, or pH 5.0 and 35°C **(B)**, and comparison with those in the influent liquor (corresponding symbols in the gray-shaded area).

The differential removal of transition metals within the aSRBR when amended, copper-free synthetic AMD was used as feed liquor during periods when it was maintained at either pH 4.0 and 30°C, or pH 5.0 and 35°C, is shown in **Figure 7**. Zinc was highly effectively removed at both pH/temperature conditions. Most of the nickel in the influent liquor was also precipitated within the bioreactor at pH 4.0 and 30°C, but this was not the case with cobalt. However, increasing the operating pH of the aSRBR to 5.0 and the temperature to 35°C resulted in the effective removal of all transition metals that were present in the influent liquor, with the exception of manganese, within the bioreactor vessel (**Table 2**). During the full course of this experiment, 5.6 g of CoS, 23.1 g of NiS, and 1.9 g of ZnS were estimated to have accumulated within the aSRBR. Off-line treatment of the 2,025 L of synthetic mine water would also have generated 1.45 kg of CuS product.

**Table 2:** Removal of transition metals within the sulfidogenic bioreactor when operated at pH 4.0 and 30°C, or at pH 5.0 and 35°C (mean values ± standard deviations, as percentages).

pH/T	Metal			
	
	Co	Mn	Ni	Zn
4.0/30°C	84.5 ± 10.5	2.3 ± 3.5	77.2 ± 12.8	92.3 ± 24.2
5.0/35°C	90.9 ± 6.8	7.0 ± 4.4	94.2 ± 8.2	94.7 ± 16.7


### Chemical and Microbiological Analysis of the Biofilm Bed

**FIGURE 8:**
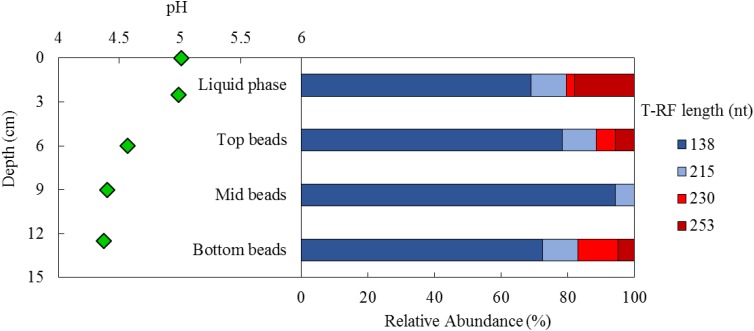
Depth-related variations in pH **(Left)** and T-RFLP profiles of the bacterial community (HaeIII digests of amplified 16S rRNA genes) of planktonic cells (liquid phase) and colonized beads (top, mid, bottom bead layers) **(Right)** within the aSRB reactor, when maintained at pH 5.0 and 35°C. Shades of blue represent sulfate-reducing *Firmicutes* (138 nt, *Peptococcaceae* strain CEB3 and 215 nt, *D. acididurans*), shades of red represent non-sulfidogens (230 nt, *Actinobacterium* AR3 and 253 nt, *A. ferrooxidans*).

Samples taken from the biofilm bed at the end of the test period, when the aSRBR was maintained at pH 5.0 and 35°C, indicated that most of the acidity of the influent liquor (pH 2.1) had been neutralized within a short distance of its upward passage through the biofilm bed (**Figure 8**). The measured pH of the interstitial water in the bed 2–3 cm above the entry point was 4.3, and this continued to increase as the influent liquor migrated through the bed into the uniformly pH 5.0 surface liquid later.

**FIGURE 9:**
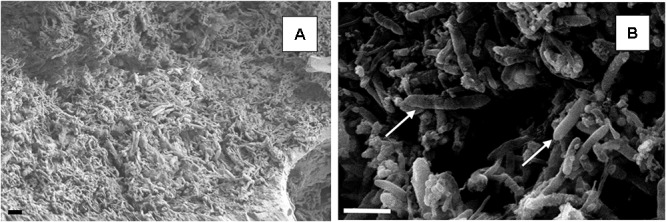
Scanning electron micrographs of colonized beads within the biofilm bed of the aSRBR. The scale bar represents 3 μm in both images. Low magnification image **(A)**. Rod-shaped bacteria, typical of aSRB *Firmicutes*, are arrowed in **(B)**.

**FIGURE 10:**
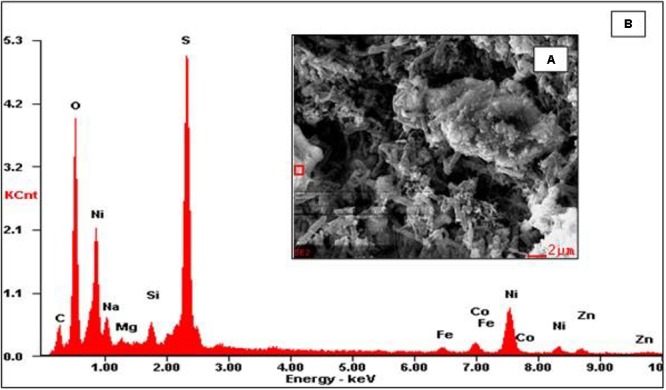
Micro-analysis of biofilm-colonized glass beads by electron dispersive analysis of X-rays (EDAX). **(A)** SEM image displaying selected area (red square) and **(B)** EDAX spectrum of the highlighted area.

Scanning electron micrographs confirmed that the porous glass beads in the aSRBR had become extensively colonized by prokaryotes during the experimental test period (**Figure 9**), with bacteria similar in size and morphologies to aSRB *Firmicutes* detected by T-RFLP analysis (**Figure 8**) being very abundant. Micro-analysis of biofilm-colonized glass beads by EDAX confirmed the presence of the three transition metals (cobalt, nickel, and zinc) that had been shown to precipitate within the aSRBR, and a large peak corresponding to sulfur implicating the presence of solid-phase sulfide (**Figure 10**). Of the other elements detected by EDAX analysis, carbon and part of the oxygen were considered to derive from the biomass present, silicon, sodium, and most of the oxygen from the silicaceous beads, and iron from corrosion products of the metallic components of the bioreactor during the long-term trial operated under chemically aggressive conditions.

Repeated attempts to amplify archaeal 16S and eukaryotic 18S rRNA genes from liquid and biofilm bead samples all proved negative. The microbial community within the aSRBR was therefore assumed to be entirely bacterial. Community profiles of beads taken from different depths within the biofilm bed and of the surface liquid layer, as revealed by T-RFLP analysis, are shown in **Figure 8**. In all cases, the dominant bacteria were aSRB *Firmicutes* (*Peptococcaceae* strain CEB3 and *D. acididurans*); known non-sulfidogens (*Acidithiobacillus ferrooxidans* and *Actinobacterium* strain AR3) were also detected in the liquid phase and two of the three biofilm bead samples.

## Discussion

Active biological treatment of mine waters using sulfate-reducing bioreactors has the potential to radically improve the ways by which this form of environmental pollution is mitigated. There are numerous advantages to be gained by operating a sulfidogenic bioreactor at low pH, as opposed to circum-neutral pH, as is currently the case. These include: (i) acidic waters can be treated directly within the bioreactor module, which reduces engineering complexity; (ii) the main product is a gas (H_2_S), which is more readily transferred to off-line reactor vessels than soluble HS^-^ (the major product at and above pH 7); (iii) biosulfidogenesis can neutralize acidity when carried out at low pH, since the main metabolic waste products are H_2_S and H_2_CO_3_, rather than HS^-^ and HCO_3_^-^ (10-times more hydronium ions are consumed by sulfate reduction at pH 4 than at pH 7); (iv) transition metals can be both precipitated off-line and selectively precipitated *in situ*, by operating a bioreactor at a designated pH value. Sulfate (though not sulfur) reduction also facilitates the removal of anionic sulfate from waste waters, which is a primary objective is some circumstances (e.g., Ñancucheo and Johnson, 2014).

In the present study, a synthetic version of a moderately acidic waste water generated at a working copper mine in Brazil was targeted both for remediation and metal recovery using a single newly commissioned, low pH sulfidogenic bioreactor. Rates of sulfate reduction (14.8 ± 2.1 mmoles L^-1^ day^-1^ when the bioreactor was operated at pH 5.0 and 35°C) were comparable to those reported previously for low pH sulfidogenic bioreactors (e.g., ∼21 mmoles L^-1^ day^-1^, Ñancucheo and Johnson, 2014; ∼3–16 mmoles L^-1^ day^-1^, Santos and Johnson, 2017). The different scales of concentrations of copper and the other transition metals present in the synthetic mine water meant that the former could be readily removed and recovered as a sulfide phase while other metals needed to be concentrated ahead of being recovered. Removal of copper was achieved off-line using excess H_2_S generated by the aSRBR. The large generation of acidity that accompanied the formation of copper sulfide precluded the formation of other metal sulfides (the remaining transition metals have larger solubility products for their sulfide phases which means that they would not form below pH 3; Monhemius, 1977), and also allowed the partly processed water to be suitable for use as a feed for the aSRBR. Removal of most of the other transition metals (cobalt, nickel, and zinc) was achieved by allowing them to accumulate as sulfide phases (confirmed by EDAX analysis) within the bioreactor vessel. The experiment was terminated after 15 months not because of any downturn in bioreactor performance, but due to the requirement to sample the biofilm bed in a semi-destructive manner, and to explore ways to remobilize metals that had accumulated within the aSRBR. This was demonstrated to be feasible by changing the bioreactor to an aerobic system, gassing with air rather than nitrogen, dropping the pH to ∼1.5 and inoculating with a sulfide mineral-oxidizing consortium (Rawlings and Johnson, 2007; and unpublished data).

All of the transition metals present in the synthetic AMD were removed, either off-line or in-line, with the notable exception of manganese. This metal has a much larger sulfide solubility product than the other transition metals present, and MnS consequently only forms at pH values well above 7. Soluble manganese (II) is more effectively removed by facilitating its oxidation to highly insoluble manganese (IV), which can be achieved either chemically or biologically (Mariner et al., 2008; Bohu et al., 2016).

Although ∼7 mM sulfate was consumed within the bioreactor, its concentration in the effluent liquor generated (∼19 mM) was actually greater than that in water at the Sossego mine water (15.7 mM). A major reason for this is that the copper-free synthetic mine water was amended with a basal salts mixture, which included ∼6 mM combined ammonium and magnesium sulfates, at normal culture dosing rates. Addition of nitrogen, phosphorus, and other inorganic nutrients were well in excess of that required (as illustrated below) and could be lowered considerably and have no negative impact on the indigenous microbial biomass, and with net beneficial impact on the quality of the waste water generated by the aSRBR.

Numbers of microbial cells in the effluent liquors were typically ∼6 × 10^7^ mL^-1^. Assuming steady state (i.e., numbers of cells washed out were balanced by new biomass, as indicated by other performance indicators) it is possible to estimate the minimum requirement of nitrogen and phosphorus (as well as other macronutrients) to sustain the growth of the bacteria. Assuming the dry weight of a single bacterium to be ∼2 × 10^-13^ g, and that this contains 15% (by wt.) N and 3% P (Perry et al., 2002), the minimum amounts required would be 130 μM N and 11 μM P, whereas the basal salts amendment would have provided 3.4 mM and 370 μM of these, respectively, implying that a 90% reduction in supply of both of these macronutrients could be contemplated.

Sulfate added to the aSRBR is both assimilated and dissimilated, the relative proportions of which can be calculated using a similar approach to that above, and assuming that the bacterial cells contain ∼1% (dry wt.) of sulfur. On that basis, the amount of biomass sulfur washed out of the aSRBR is ∼0.12 mg L^-1^ (3.75 μM), which is a tiny fraction of the sulfate typically consumed (∼7 mM). It was therefore concluded that >99.9% of the sulfate removed in the aSRBR was reductively dissimilated, principally to H_2_S.

The role of glycerol in the aSRBR is potentially more complex. Like sulfate some would have been assimilated though this is again considered to be a minor fraction, especially since yeast extract would have probably been a significant carbon source for the indigenous microflora. The microbial consortium in the aSRBR (described below) contains bacteria that can ferment glycerol, as well as those that use it as an electron donor coupled to sulfate reduction. The relative contributions of these two groups to net glycerol catabolism were estimated from mass balance calculations, which took into account the stoichiometry of glycerol oxidized to sulfate reduced when the former is oxidized completely to CO_2_ (4:7) and when it is incompletely oxidized to acetate + CO_2_ (4:3), and measurements of net acetate production (**Figure 5**). On that basis, there was very close agreement between glycerol oxidized and sulfate reduced (**Figure 6**) which was supported by results from T-RFLP analysis, which showed that sulfate-reducing bacteria dominated the community profiles of both planktonic and attached (biofilm) populations.

Measurements of pH of samples taken from the aSRBR indicated that most of the acidity of the influent liquor was rapidly neutralized as it migrated through the biofilm bed. The influent liquor had a pH of 2.1 mM, which corresponds to 8 mM hydronium ions. Bisulfate (HSO_4_^-^) would have contributed an additional 10.3 mM acidity and the soluble transition metals (Co, Ni, and Zn) 0.62 mM, so that the influent liquor contained 18.92 mM total acidity. At pH 4.3 (the value in the biofilm bed at the lowest depth sampled) the residual total acidity (assuming the three transition metals had precipitated as sulfides) is only 0.05 mM, i.e., >99% of the total acidity was seemingly neutralized by this stage of passage though the biofilm bed. This probably overestimates the rapidity of the change, however, as there would have been some movement of dissolved solutes within interstitial liquors in the biofilm caused both by diffusion and mass flow (due by agitation induced by gas flow). Superficially, since the pH of the effluent generated by the aSRBR was the same as that of the synthetic AMD (5.0), it would appear that there was no amelioration of the acidity of the mine water in this process. However, taking into account the total mineral acidity (the summated concentrations of soluble transition metals) which was 16.32 mM in the synthetic AMD, and 0.31 mM (due mostly to the presence of residual soluble manganese) in the processed water, it can be seen that 98% of the total acidity (in addition to removal of 98% of the total transition metal load) was removed using sulfate-reducing biotechnology.

Four bacterial species were detected by T-RFLP analysis in the aSRBR. All four had also been reported previously to be present in similar modules that had been inoculated with the same “mother reactor” culture (Ñancucheo and Johnson, 2012, Johnson, 2014; Santos and Johnson, 2017). Two of these are known acidophilic sulfate-reducing *Firmicutes*. *Peptococcaceae* strain CEB3, which was the most abundant microorganism in all layers of the biofilm bed and the surface liquid in the aSRBR, is a partially characterized sulfidogen of the proposed genus “*Desulfobacillus*” (Dopson and Johnson, 2012) which is able to oxidize glycerol completely to carbon dioxide, and is thought to be the acid-tolerant SRB in the consortium used (Santos and Johnson, 2017). *D. acididurans*, which was added to the consortium as a pure culture of its type strain, M1 (Sánchez-Andrea et al., 2015) oxidizes glycerol incompletely, generating stoichiometric concentrations of acetate (Kimura et al., 2006), and was thought to be the major source of this aliphatic acid in the effluent liquor. *Actinobacterium* strain AR3 is an obligately heterotrophic bacterium that has been isolated from the culture and shown to grow on glycerol both under anaerobic (presumably by fermentation) and micro-aerobic conditions, again generating acetate. *A. ferrooxidans* is the most widely studied of all acidophilic bacteria, and can use reduced sulfur, ferrous iron, and hydrogen as electron donors, and molecular oxygen or ferric iron as electron acceptors. Given the lack of availability of some of these in the aSRBR, its most likely mode of metabolism in the bioreactor is coupling the oxidation of hydrogen sulfide to the reduction of trace amounts of oxygen present in the influent liquors, which were not de-aerated. It is interesting in that respect that *A. ferrooxidans* was found in the biofilm bed at both the bottom and the surface of the aSRBR vessel, where it would have had greater access to oxygen present in the feed liquors and possibly diffusing in through the seals in the top plate, but not in the mid-beads sample, which were probably devoid of oxygen. The absence of methanogenic (and other) archaea, and fermentative eukaryotes (e.g., yeasts) minimized competition for electron donors and helped optimize the efficiency of the aSRBR module in generating hydrogen sulfide.

This work has demonstrated how a single low pH sulfidogenic bioreactor can be used to remediate a metal-rich mine water, and to facilitate the recovery (and therefore recycling) of target metals. The system was shown to be robust, and could be operated empirically by means of pH control. In terms of operating costs (OPEX), it was estimated that, to treat 100 m^3^ of mine water, ∼46 kg of glycerol and 10 kg of yeast extract (commodity prices quoted in May 2018: $ 0.88 kg^-1^ and $ 2.00 kg^-1^, respectively) would be required as electron donor and carbon sources, giving a combined OPEX of ∼$ 60.50. In terms of metal products, the combined values of copper, cobalt, nickel, and zinc is estimated to be $ 365.00 (metal prices quoted in May 2018). Other OPEX costs (e.g., inorganic nutrients and flow gas) and capital-investment (CAPEX) costs also need to be taken into account, as do costs involved in the alternative chemical treatment and sludge disposal approach currently in place. However, together both the economic and environmental/sustainability benefits associated the applying sulfidogenic biotechnology in this (as in many other situations) are highly attractive.

## Author Contributions

AS contributed to the experimental work, writing the manuscript, and preparing the tables and figures. BJ wrote the manuscript and designed the experiments.

## Conflict of Interest Statement

The authors declare that the research was conducted in the absence of any commercial or financial relationships that could be construed as a potential conflict of interest. The reviewer MD declared a past collaboration with one of the authors BJ to the handling Editor.
